# Other PHAC publications

**DOI:** 10.24095/hpcdp.44.2.07

**Published:** 2024-02

**Researchers from the Public Health Agency of Canada also contribute to work published in other journals and books. Look for the following articles published in 2023:**

**Bird M**, Barnett TA, Fuller D, et al. Multidimensional school features associated with physical activity among youth at risk of obesity: an exploratory principal component and generalized estimating equation analysis. BMC Public Health. 2023;23(1):2010. https://doi.org/10.1186/s12889-023-16889-w

Fuller-Thomson E, Dolhai H, MacNeil A, […] **Jiang Y, de Groh M**. Depression during the COVID-19 pandemic among older Canadians with peptic ulcer disease: analysis of the Canadian Longitudinal Study on Aging. PLoS ONE. 2023;18(10):e0289932. https://doi.org/10.1371/journal.pone.0289932

**Lavergne V, Butler G, Prince SA, Contreras G**. Associations between school-level environment and individual-level factors of walking and cycling to school in Canadian youth. Prev Med Rep. 2023;36:102489. https://doi.org/10.1016/j.pmedr.2023.102489 

Poon ET, Tomkinson GR, **Lang JJ**, et al. Temporal trends in the physical fitness of Hong Kong children aged 6–12 years between 2003–04 and 2015–16. J Sports Sci. 2023;41(13):1271-8. https://doi.org/10.1080/02640414.2023.2268350

**Varin M, Champagne A, Venugopal J, Li L, McFaull SR, Thompson W, Toigo S, Graham E, Lowe AM**. Trends in cannabis-related emergency department visits and hospitalizations among children aged 0–11 years in Canada from 2015 to 2021: spotlight on cannabis edibles. BMC Public Health. 2023;23(1):2067. https://doi.org/10.1186/s12889-023-16987-9

